# Effects of Selective and Non-Selective Glucocorticoid Receptor II Antagonists on Rapid-Onset Diabetes in Young Rats

**DOI:** 10.1371/journal.pone.0091248

**Published:** 2014-03-18

**Authors:** Jacqueline L. Beaudry, Emily C. Dunford, Trevor Teich, Dessi Zaharieva, Hazel Hunt, Joseph K. Belanoff, Michael C. Riddell

**Affiliations:** 1 School of Kinesiology and Health Science, Faculty of Health, Muscle Health Research Center and Physical Activity and Chronic Disease Unit, York University, Toronto, Ontario, Canada; 2 Corcept Therapeutics, Menlo Park, California, United States of America; University of Ulm, Germany

## Abstract

The blockade of glucocorticoid (GC) action through antagonism of the glucocorticoid receptor II (GRII) has been used to minimize the undesirable effects of chronically elevated GC levels. Mifepristone (RU486) is known to competitively block GRII action, but not exclusively, as it antagonizes the progesterone receptor. A number of new selective GRII antagonists have been developed, but limited testing has been completed in animal models of overt type 2 diabetes mellitus. Therefore, two selective GRII antagonists (C113176 and C108297) were tested to determine their effects in our model of GC-induced rapid-onset diabetes (ROD). Male Sprague-Dawley rats (∼ six weeks of age) were placed on a high-fat diet (60%), surgically implanted with pellets containing corticosterone (CORT) or wax (control) and divided into five treatment groups. Each group was treated with either a GRII antagonist or vehicle for 14 days after surgery: CORT pellets (400 mg/rat) + antagonists (80 mg/kg/day); CORT pellets + drug vehicle; and wax pellets (control) + drug vehicle. After 10 days of CORT treatment, body mass gain was increased with RU486 (by ∼20% from baseline) and maintained with C113176 administration, whereas rats given C108297 had similar body mass loss (∼15%) to ROD animals. Fasting glycemia was elevated in the ROD animals (>20 mM), normalized completely in animals treated with RU486 (6.2±0.1 mM, p<0.05) and improved in animals treated with C108297 and C113176 (14.0±1.6 and 8.8±1.6 mM, p<0.05 respectively). Glucose intolerance was normalized with RU486 treatment, whereas acute insulin response was improved with RU486 and C113176 treatment. Also, peripheral insulin resistance was attenuated with C113176 treatment along with improved levels of β-cell function while C108297 antagonism only provided modest improvements. In summary, C113176 is an effective agent that minimized some GC-induced detrimental metabolic effects and may provide an alternative to the effective, but non-selective, GRII antagonist RU486.

## Introduction

Glucocorticoids (GCs) are naturally occurring steroid-derived hormones that are essential for healthy whole-body metabolism and adaptation to stressful environments. The hypothalamic-pituitary-adrenal (HPA) axis is the main regulator of GC secretion (cortisol in humans and corticosterone in rodents), operating normally in a diurnal rhythm and in response to stressors to increase GC release [1]. GCs act as ligands and bind to receptors including GC receptors II (GRII) that are found ubiquitously throughout the body. The mineralocorticoid receptor (MR) also binds GCs with high affinity [2], but expression of this receptor is lower than GRII in most tissues except for in the hippocampus, kidneys, adipose tissue and heart [3] and plays a larger role in non-stressful conditions. Once the GRII is occupied by a ligand, the complex translocates to the nucleus where it acts as a transcription factor to activate or repress the expression of genes necessary for cell proliferation, inflammation, immune development, reproduction [4] and energy homeostasis [5], [6].

Acute elevations in GCs are important for many biological functions; however, chronically high levels of GCs, such as those observed in patients suffering from Cushing's syndrome [7], result in unwanted metabolic disturbances such as attenuated lean tissue growth [8], increased whole body insulin resistance [9], elevated fasting glucose levels [10] and increased risk for type 2 diabetes mellitus (T2DM) development [11]–[13]. Currently, there are a large number of rodent [14]–[16] and human [17], [18] studies suggesting that the rise in circulating and/or cellular levels of GCs is connected with diabetes onset. At the cellular level, the pre-receptor enzyme, 11β-hydroxysteroid dehydrogenase type 1 (11β-HSD1) is responsible for conversion of inactive GCs into active GCs. This activity increases GC concentrations leading to the progression of tissue specific metabolic dysfunction [19] and, if left untreated, T2DM development [20]. GRII antagonists have become an active area of interest as they may eliminate unwanted metabolic effects of elevated GCs.

Mifepristone (RU486) is a non-selective GRII antagonist that competitively blocks the GRII, the progesterone receptor (PR), but not the MR [21]. This receptor antagonist has recently gained FDA approval (Korlym™, 2012) for the treatment of patients with hypercortisolemia or Cushing's syndrome with established hyperglycemia, since it has been shown clinically to improve glucose tolerance in these patients [22]–[24]. However, RU486 treatment requires consistent patient monitoring as it can result in various side effects such as endometrial hypertrophy, hypokalemia, and aborted pregnancy [21], [25]. Currently, more specific antagonists selective for the GRII are being developed in the hope of eliminating the progesterone blocking properties of RU486 administration. A few studies of selective GRII antagonists have been conducted in rodent models [26]–[28]. In these studies, selective GRII antagonists have been shown to play a role in the attenuation of detrimental GRII-dependent pathways in the brain [26], whole-body steady state glucose metabolism [27], and body mass gain [28]. However, no studies in this field have been conducted to investigate the role of selective GRII antagonists in a model of elevated GCs indicative of Cushing's syndrome and diabetes development.

Recently, we have developed a rodent model of rapid-onset diabetes (ROD) that involves the administration of increased levels of GCs by a slow release corticosterone pellet in combination with a high-fat diet (HFD) in young male Sprague-Dawley rats [29], [30]. We are interested in the effects of GRII antagonists on ROD and hypothesized that this therapeutic treatment will help to prevent ROD development. In this present study, we tested two selective GRII antagonists, C113176 and C108297, in comparison to the non-selective GRII antagonist, RU486, in our established rodent model of ROD. We show that a new selective GRII antagonist, C113176, has beneficial effects in our model of ROD, attenuating the pathophysiological outcomes of elevated GCs and high-fat feeding, including perturbed body composition, elevated fasting glucose concentrations, insulin resistance and reduced insulin release by pancreatic β-cells.

## Materials and Methods

This study was carried out in accordance with the recommendations of the Canadian Council for Animal Care guidelines and was approved by the York University Animal Care Committee (Protocol # 2010-15(R2)). All surgical procedures were performed under isoflurane anaesthesia, and all efforts were made to minimize animal suffering.

### Rodent Treatment and Experimental Design

Three sets of 20 male Sprague Dawley rats (Charles River Laboratories, 225–250 g, six weeks post-weaned) were individually housed (lights on 12 h: lights off 12 h cycle) after one week of acclimatization to the rodent facilities. Upon surgery day (day 0), each rat received a subcutaneous implantation of either corticosterone (CORT) pellets (4×100 mg; Sigma, Canada, Cat # C2505) or wax (control) pellets, as previously described [29]. Immediately following surgery all rats were given *ad libitum* access to a high-fat diet (HFD), 60% of the total calories from fat and 5.1 kcal per gram of pellet (D12492, Research diets); this diet was maintained to the end of the experimental protocol. Rats recovered in sterile cages for two days and were then separated into five groups while being maintained on a HFD and were assigned either vehicle or GRII antagonist treatment (RU486, C113176 and C108297) at a dose of 80 mg/kg/day. These antagonists have been partially characterized with respect to binding efficiencies to GR, MR, PR, androgen receptor (AR), and estrogen receptor (ER) in human HepG2 and Rat H4 cells (Table 1). The compounds RU486, C113176 and C108297 have been shown to readily distribute into liver, adipose tissue, and brain tissue and to have similar pharmacokinetic profiles (Communications with Corcept Therapeutics). The antagonist dose was chosen based on doses used in earlier studies [27]. The following treatment groups were created; wax+ vehicle gavage controls (controls), CORT+vehicle (ROD), CORT+RU486 (RU486), CORT+C108297 (C108297), CORT+C113176 (C113176). All antagonists were first dissolved in DMSO which was further dissolved in a vehicle consisting of 0.5% HPMC + 0.1% Tween 80 by approximately 30 seconds of sonication. ROD animals who were given only vehicle received 10% DMSO + vehicle instead of an antagonist compound. Antagonists and vehicles were administered with a syringe and oral gavage tube (Instech, Plymouth, PA USA, Cat # FTP-18-75) twice daily, approximately 10 hours apart. Body mass and food intake were measured and recorded daily for each rodent using an electronic scale (Mettler Toledo, Canada) and any changes in the rodents' health were noted and monitored.

**Table 1:** Binding affinities (Ki) and TAT activity assay data for RU486, C108297 and C113176 to GR, MR, PR, AR, and ER in hepatocytes.

	Compounds		
	RU486	C108297	C113176
**GR**	0.09 nM	0.28 nM	0.26 nM
**MR**	5.5 µM	0% @ 10 µM	20% @ 10 µM
**PR**	1 nM	26% @ 10 µM	0% @ 10 µM
**AR**	15 nM	12% @ 10 µM	2% @ 10 µM
**ER**	392 nM	3% @ 10 µM	37% @ 10 µM
**Human HepG2**	3 nM	25 nM	11 nM
**Rat H4**	2 nM	14 nM	4 nM

Note: The Ki in human HepG2 and rat H4 cells were calculated based on the IC50 data from TAT assay using dexamethasone treatment. Data provided by Corcept Therapeutics Incorporated.

### Blood Sampling

Plasma CORT levels were sampled on the morning of the 7^th^ and 14^th^ day after pellet implantation at approximately 0800 h, using the animal's saphenous vein. Blood samples were collected in lithium-heparin coated microvette capillary tubes (Sarstedt, Des Grandes Prairies, Montreal, Québec, Canada, Cat # 16.443.100) and centrifuged at 12,000 rpm for 5 minutes so that the plasma could be collected into polyethylene tubes and stored at −80°C until further analysis. These samples were later analyzed for CORT levels using a radioimmunoassay kit (MP Biomedical, OH, USA).

Fed whole blood glucose concentrations were measured on day seven using a handheld glucometer (Bayer, Contour, NY, USA). On day 11, animals were fasted overnight (16 hours) and on day 12, animals were administered an oral glucose tolerance test (OGTT, 1.5 g/kg body mass). An insulin tolerance test (ITT) was administered on day 16 after an overnight fast by intraperitoneal (i.p.) insulin injection, (as described in [29], [30]). For these tests, all blood glucose concentrations were also measured with a handheld glucometer and blood samples drawn from saphenous vein were collected in microvette tubes (Sarstedt). The animals' plasma was subsequently analyzed for insulin (Crystal Chem, IL, USA, Cat # 90060) using the high-range assay method and for non-esterified fatty acids (NEFA) levels (HR Series NEFA-HR, Wako Chemicals). Glucose area under the curve (AUC) and insulin AUC was measured relative to each individual's fasting insulin levels (i.e. each groups baseline) to assess the groups' responsiveness to oral glucose challenge. The acute insulin response (AIR) to oral glucose challenge was determined by the difference between basal (fasting) insulin levels and insulin levels 15 minutes following an oral glucose gavage (as described in [30], ). This measurement represents the ability of the pancreatic β-cells to respond to exogenous glucose load. The glucose AUC measured during the ITT was determined by the net inverse relationship between the individual fasting and 30-minute blood glucose post insulin injection.

Homeostatic Model Assessment for Insulin Resistance (HOMA-IR) was calculated as previously reported in [29] and is based on the following equation: Glucose (mM)×Insulin (μ units·L)/22.5 [32]. This calculation represents basal glucose and insulin action on peripheral tissues, and primarily reflects the relationship between hepatic glucose output and insulin secretion [33]. Homeostatic Model Assessment for β-cells (HOMA-β) was calculated based on the following equation: 20×Insulin (μ units·L)/Glucose (mM)-3.5 [32]. This measurement represents basal pancreatic β-cell function in response to basal glucose levels.

For technical and experimental reasons, the day of termination ranged from 2–5 days after the ITT to allow for subsequent tissue collection. Trunk blood was collected for further analysis. Tissues collected from animals euthanized via decapitation were as follows: liver, heart, epididymal fat pads, and skeletal muscles such as epitrochlearis, soleus, gastrocnemius and tibialis anterior.

### Histology

Liver and skeletal muscle tissue from euthanized animals were snap frozen, cryosectioned (10 µm thick) and stained with Oil Red O for neutral lipid content as previously described [34]. Muscle and liver sections were fixed with 3.7% formaldehyde for 1 h at room temperature while an Oil Red O solution composed of 0.5 g Oil Red O powder (Sigma-Aldrich, Canada) and 100 ml of 60% triethyl phosphate (Sigma-Aldrich, Canada) was mixed and filtered. Following fixation in 3.7% formaldehyde, slides were immersed in filtered Oil Red O solution for 30 minutes at room temperature. Slides immediately underwent five washes with ddH2O, were allowed to dry for 10 minutes and were sealed with Permount (Sigma-Aldrich, Canada). Skeletal muscle and liver images were acquired at 10× and 20× magnifications respectively using a Nikon Eclipse 90i microscope (Nikon, Canada) and Q-imaging MicroPublisher 3.3 RTV camera with Q-capture Software. Intensity of Oil Red O staining of IMCL droplets on serial sections of the tibialis anterior was assessed with Adobe Photoshop CS6, converted to greyscale and reported as the average optical density (60 fibers were counted per muscle section). The greyscale is evaluated on a range of 0 (completely black) to 255 (completely white).

### Western Blotting

We quantified protein expression for key determinants of adipose tissue metabolism including CD36, adipose tissue triglyceride lipase (ATGL), hormone sensitive lipase (HSL) and 11β-HSD1. Western blot analysis was carried out according to previously published work [30], [35]. In brief, 50 µgs of protein lysate from epididymal fat protein was run on a 10% (CD36, ATGL, HSL) or 12% (11β-HSD1) SDS-page gel and proteins were transferred to a PVDF membrane (Bio-Rad, Canada). Membranes were blocked in 10% powdered milk and Tris-buffered saline with Tween 20 at room temperature for 1 hour. Membranes were then incubated overnight at 4°C with their respective primary antibodies (CD36, 1∶1000, ab133625, Abcam, Toronto, ON; ATGL, 1∶500, sc-50223, Santa Cruz Biotechnology, Dallas, TX; HSL, 1∶1000, sc-25843, Santa Cruz Biotechnology; 11β-HSD1, 1∶1000, Cat#10004303, Cayman Chemical Company, Ann Arbor, MI;). The following morning the membranes were washed with TBST and incubated with anti-mouse (1∶10000, Cat#ab6789, Abcam) or anti-rabbit (1∶10000, Cat#ab6721, Abcam) secondary antibodies for 1 hour at room temperature. Membranes were then washed and imaged. Images were detected on a Kodak In vivo FX Pro imager and molecular imaging software (Carestream Image MI SE, version S.0.2.3.0, Rochester, New York) was used to quantify protein content. To minimize reprobing due similar molecular weights, α-tubulin (1∶40000, ab7291, Abcam) was used as a loading control for 11β-HSD1 and HSL, and β-actin (1∶20000, ab6276, Abcam) was used for CD36 and ATGL.

### Glucose Stimulated Insulin Secretion (GSIS)

Islet isolations and *ex vivo* glucose challenges were carried out as previously reported [30]. Collagenase pancreas digestion was followed by Histopaque-1077 (H8889, Sigma, Canada) pellet suspension followed by re-suspension in KREB's buffer. Islets were handpicked and cultured in filtered RPMI buffer (Wisent) overnight (24 h) at 37°C, 5% CO_2_. Islets were separated into a 12-well culture plate (6–10 islets/well in 3 batches) and given a 30-minute pre-incubation period as previously described [30]. Islets were given fresh KREB's buffer with 2.8 mM glucose + 0.1% BSA for 1 hour at 37°C, 5% CO_2_. Media was changed to KREB's buffer with 16.7 mM glucose + 0.1% BSA for 1 hour at 37°C, 5% CO_2_. Immediately following each incubation period, media was collected, centrifuged and stored at −20°C for further analysis. Insulin was measured using a radioimmunoassay kit (Millipore, Billerica, MA, USA).

### Statistical Analysis

All data are represented as a mean ± standard error (SE), with a criterion of p<0.05 and were assessed using one-way ANOVAs as a means of statistical significance. All individual differences were evaluated using Tukey's post-hoc test unless otherwise stated as a student's t-test (Statistica 6.0 software and Prism Graph Pad version 5.1). Results from post-hoc analyses were denoted on each figure bar using letters. If bars do not share the same letters then mean values were found to be statistically significant between treatment groups. If bars share the same letter then mean values were found to not be statistically significant from each other.

## Results

### Body Mass

**Figure 1:**
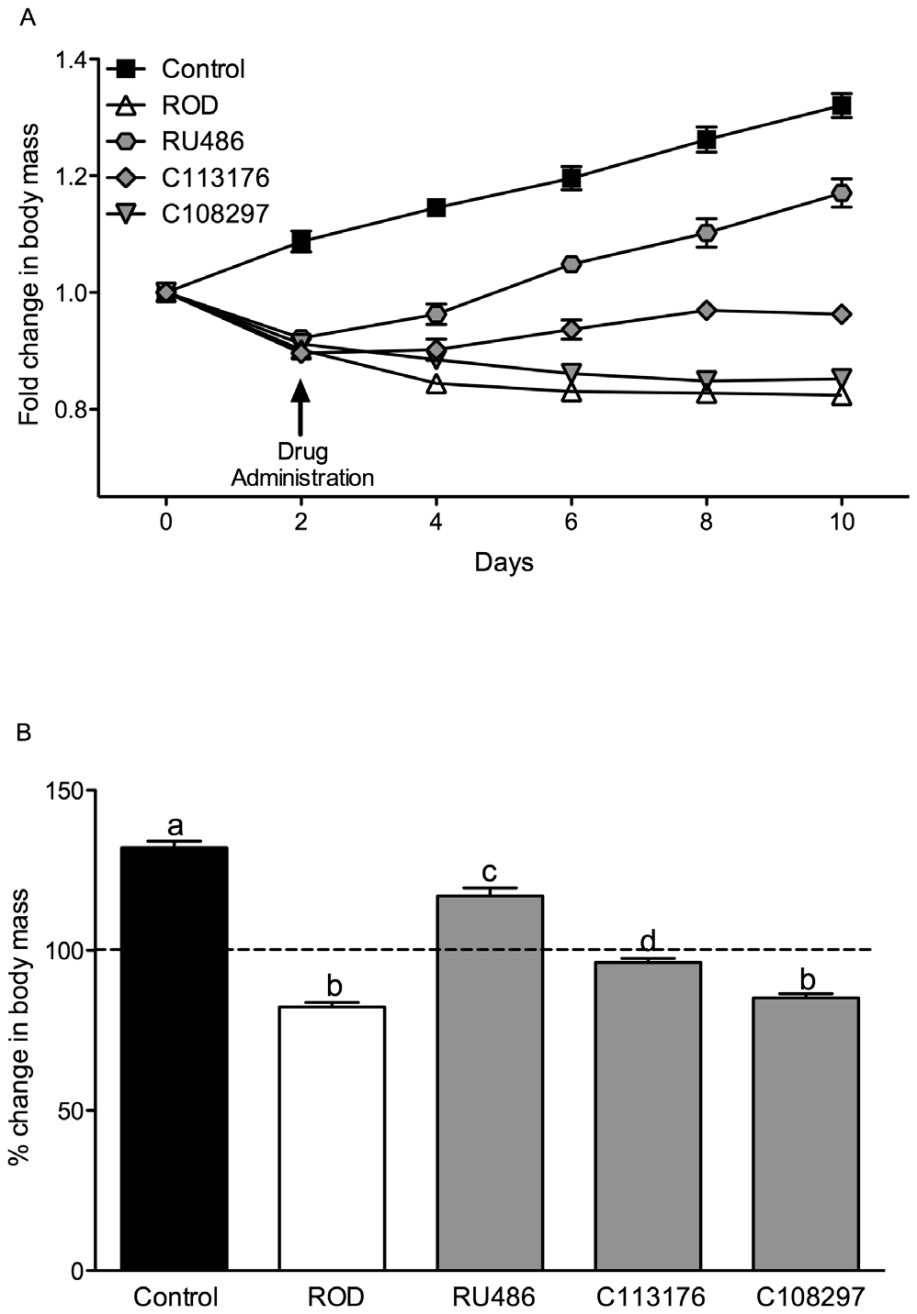
C113176 treatment maintains body mass while RU486 increases body mass with ROD treatment. Animal body mass (g) were recorded every two days for 10 days as a measure of fold change from day 0, pellet surgery (A). Animal body mass on day 10 was measured as a percent change of body mass from day 0 (B). The dotted line (100%) represents no change in body mass from day 0. Arrow indicates that 2 days after pellet surgeries respective antagonists or vehicle were administered at 80 mg/kg/day to each treatment group. Bars that do not share similar letters denote statistical significance, p<0.05, one-way ANOVA using Tukey's post-hoc test. n = 7–10. All values are means ± SE.

GC antagonist treatment commenced on day 2 of pellet treatment and body mass was measured every day relative to day 0 (pre-surgery) for 10 days. Animals weighed ∼300–325 g prior to pellet surgery (Figure 1 shows the fold changes in mass over the 10 day period). ROD treatment resulted in ∼15% body mass loss relative to pre-surgery mass and ∼50% body mass difference compared to control animals 10 days after CORT treatment. In comparison, RU486 treatment increased body mass gain relative to ROD treated animals by day 4. Body mass continued to rise during the treatment period and ultimately resulted in ∼20% mass gain over the 10 day period. It is suspected that because of the initial mass lost from day 0 to 2, RU486 treatment did not fully reverse body mass loss in CORT treated animals when compared to controls (final mass on day ten: 352.7±5.9 g, vs. 397.6±6.1 g, in the RU486 treated animals and control animals respectively, p<0.05, Figure 1B). C113176 treatment recovered body mass to pre-surgery levels by day 8 (mass on day 10, 317.6±3.8 g), whereas C108297 treatment resulted in an overall mass loss of ∼15% (mass on day 10, 259.3±4.4 g), similar to the ROD animals (mass on day 10, 266.5±3.1 g, Figure 1B).

### Corticosterone and Food Intake

Normally, circulating CORT concentrations follow a diurnal pattern in rodents and fluctuate from high levels in early evening hours (i.e. peak, ∼2000 h) to low levels in early morning hours (i.e. basal, ∼0800 h). In this study, blood was sampled on day 7 and on day 14 at ∼0800 h to determine basal CORT levels. Control animals had normal basal CORT levels on day 7 and day 14, values that were about 3–4-fold lower than all other treatment groups that received CORT pellets (p<0.05, Table 2). CORT pellet groups treated with ROD, RU486, C113176 and C108297 all had similar basal CORT levels at day 7 and 14 (p>0.05, i.e. not significant, Table 2).

**Table 2:** Corticosterone concentrations, absolute and relative food intake.

	Control		ROD		RU486		C113176		C108297	
	Day 7	Day 14	Day 7	Day 14	Day 7	Day 14	Day 7	Day 14	Day 7	Day 14
**Corticosterone (ng/ml)**	169±50^a^	61±30^a^	623±38^b^	537±41^b^	611±61^b^	401±65^b^	711±63^bc^	637±58^b^	442±61^b^	344±60^b^

	Day 10	Day 10	Day 10	Day 10	Day 10
**Food Intake (g)**	20.9±0.6^a^	24.0±1.2^b*^	26.3±1.0^b^	21.1±1.3^a^	20.3±1.6^a^
**Food Intake (kcal/g/BM)**	0.23±0.4^a^	0.46±0.02^b^	0.40±0.01^c^	0.40±0.02^c^	0.40±0.03^c^

Note: BM = Body Mass. Different letters denote statistical significance, p<0.05, n = 6–10. The * indicates that a significant difference was performed by a student's unpaired t-test. All values are mean ± SE.

Food intake was measured on day 10 of pellet treatment. ROD treated animals had an increase in absolute food intake compared to controls (p>0.05, Table 2). RU486 treated animals had the highest absolute food intake compared to all other groups (p<0.05, Table 2). Animals that were treated with C113176 or C108297 had similar absolute food intake to control animals (Table 2). Daily total kilocalorie intake relative to body mass was elevated in all CORT treated animals compared to controls (p<0.05); however, relative food intake was highest in ROD animals compared to all groups (p<0.05). Relative food intake was similar between RU486, C113176 and C108297 groups. Therefore, antagonist treatment lowered relative food intake compared to ROD animals but did not normalize food intake compared to the controls.

### Hormone and Blood Analyses

Fed blood glucose concentrations were elevated in ROD animals compared to controls (p<0.05, Table 3). RU486 normalized fed blood glucose levels whereas C113176 and C108297 treated animals had similar levels compared to ROD treated animals (Table 3). All animals were fasted on the night of day 11 for ∼16 h. Fasting blood glucose concentrations, measured the following day, were highest in ROD animals by ∼4-fold compared to controls (p<0.05, Table 3). RU486 treatment normalized fasted glucose levels compared to controls, while C113176 treatment resulted in improved but not complete normalization of fasting blood glucose concentrations (Table 3). C108297 treated animals had significantly lower fasting blood glucose levels compared to ROD animals; however, they had fasting blood glucose levels that were also higher than controls and RU486 treated rats (p<0.05, Table 3). ROD, RU486, C113176 and C108297 treated rats had higher fasting insulin concentrations compared to controls (p<0.05, Table 3). However, RU486 treatment resulted in lower fasting insulin levels compared to ROD animals, or rats treated with C113176 or C108297 (p<0.05). Fasting NEFAs were measured on day 12, as high levels of free fatty acids are associated with an increased risk of diabetes [36]. ROD, C113176 and C108297 treated rats demonstrated ∼2-fold increase in fasting NEFAs compared to controls (p<0.05, Table 3). In contrast, RU486 normalized fasting NEFA concentrations to levels found in control animals.

**Table 3:** Fed and fasting glucose, insulin, and non-esterified fatty acids (NEFAs) levels on day 12.

	Control	ROD	RU486	C113176	C108297
**Fed Blood Glucose (mM)**	6.6±0.2^a^	20.6±1.4^b^	9.5±1.3^a^	20.7±1.3^b^	23.6±2.3^b^
**Fasted Blood Glucose (mM)**	4.8±0.2^a^	21.0±0.6^b^	6.2±0.1^ac^	8.8±1.6^c^	14.0±1.6^d^
**Fasted Insulin (ng/ml)**	0.85±0.16^a^	6.75±0.58^b^	3.64±0.15^c^	6.46±1.04^b^	5.22±0.73^b^
**Fasted NEFAs(mM)**	0.45±0.03^a^	1.08±0.07^b^	0.49±0.05^a^	1.10±0.15^b^	1.06±0.12^b^

Note: Different letters denote statistical significance, p<0.05, n = 6–10. All values are means ± SE.

### Glucose Tolerance and Insulin Response

**Figure 2:**
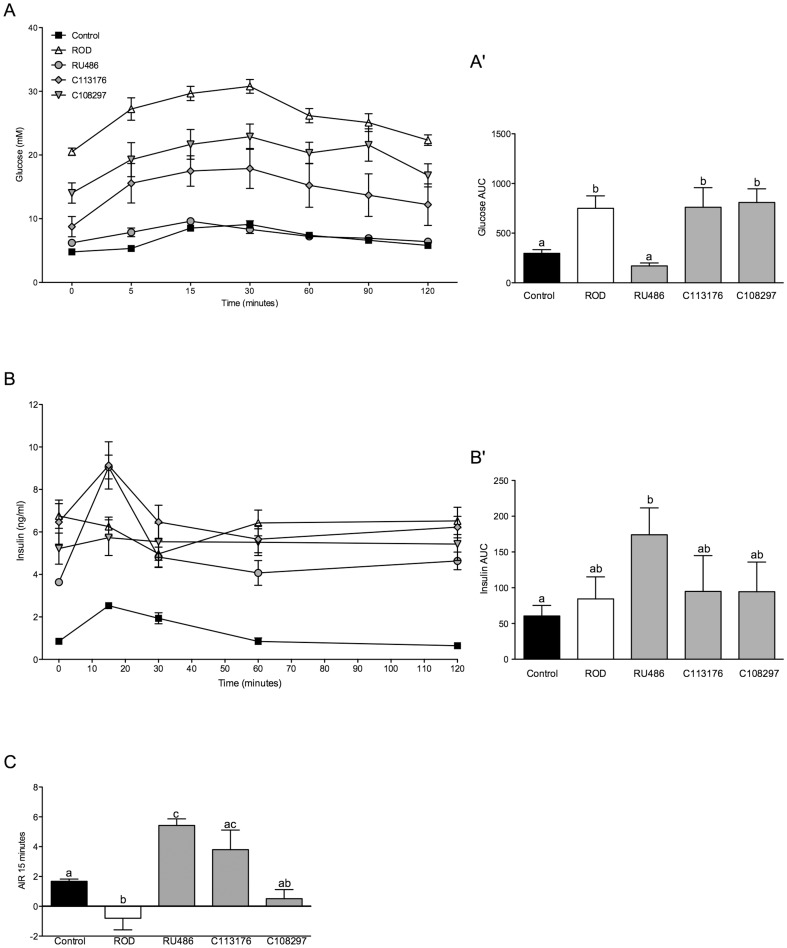
Glucose intolerance and acute insulin response (AIR) is improved with RU486 and C113176 treatment. Fasting (basal, 0 minutes) and stimulated blood glucose levels (mM) were measured at 5, 15, 30, 60, 90 and 120 minutes post oral glucose gavage (A). Glucose area under the curve (AUC) was calculated based on fasting blood glucose of individual animals (A′). Fasting (basal, 0 minutes) and glucose-stimulated insulin levels (ng/ml) were measured at 15, 30, 60, and 120 minutes post oral glucose gavage (B). Insulin area under the curve (AUC) was calculated based on fasting individual insulin levels within each group (B′). To measure insulin capacity acute insulin response (AIR) was measured by the difference in insulin levels between fasting insulin and 15 minutes post glucose gavage (C). Negative values represent a decrease in insulin response, indicating impairment in insulin secretion. Bars that do not share similar letters denote statistical significance, p<0.05, one-way ANOVA using Tukey's post-hoc. A student's unpaired t-test was performed between controls and ROD, C108297 and C113176 groups (C). n = 7–10. All values are means ± SE.

An OGTT was performed on all animals on day 12 after an overnight fast to determine glucose tolerance and insulin response to exogenous glucose challenge (Figure 2A). The ROD group had the highest glucose concentrations throughout the OGTT challenge (Figure 2A) with a glucose AUC ∼2-fold higher than controls (p<0.05, Figure 2A′). RU486 treatment normalized glucose tolerance, with results similar to those observed in the control group. C113176 treatment resulted in lower glucose levels compared to the ROD group (Figure 2A), but glucose AUC values in the animals treated with C113176 were still ∼2-fold higher than those observed in the control group (p<0.05, Figure 2A′). Treatment with C108297 also lowered glucose levels compared to the ROD treated group, but glucose AUC was still ∼2-fold higher than in control animals. All fasting and glucose-stimulated insulin levels were elevated in ROD treated animals compared to controls (p<0.05, Figure 2B). Insulin AUC was measured during the OGTT to determine *in vivo* insulin response to exogenous glucose. ROD, C113176 and C108297 treated animals had a similar AUC compared to control animals and interestingly, RU486 treatment resulted in a ∼3-fold increase in insulin AUC compared to controls (p<0.05, Figure 2B′). Acute insulin response (AIR) was measured to determine insulin response at 15 minutes post oral glucose gavage. ROD animals had reduced AIR compared to all other groups (p<0.05, Figure 2C). RU486 treatment resulted in ∼3-fold increase in AIR compared to the values in the control group (p<0.05), and C113176 treatment resulted in similar AIR levels to those observed with RU486 treatment, with higher AIR compared to animals in the control group (Figure 2C). Animals treated with C108297 showed no differences compared to control animals or ROD treated animals (p>0.05).

### Insulin Sensitivity, Insulin Resistance and β-Cell Function

**Figure 3:**
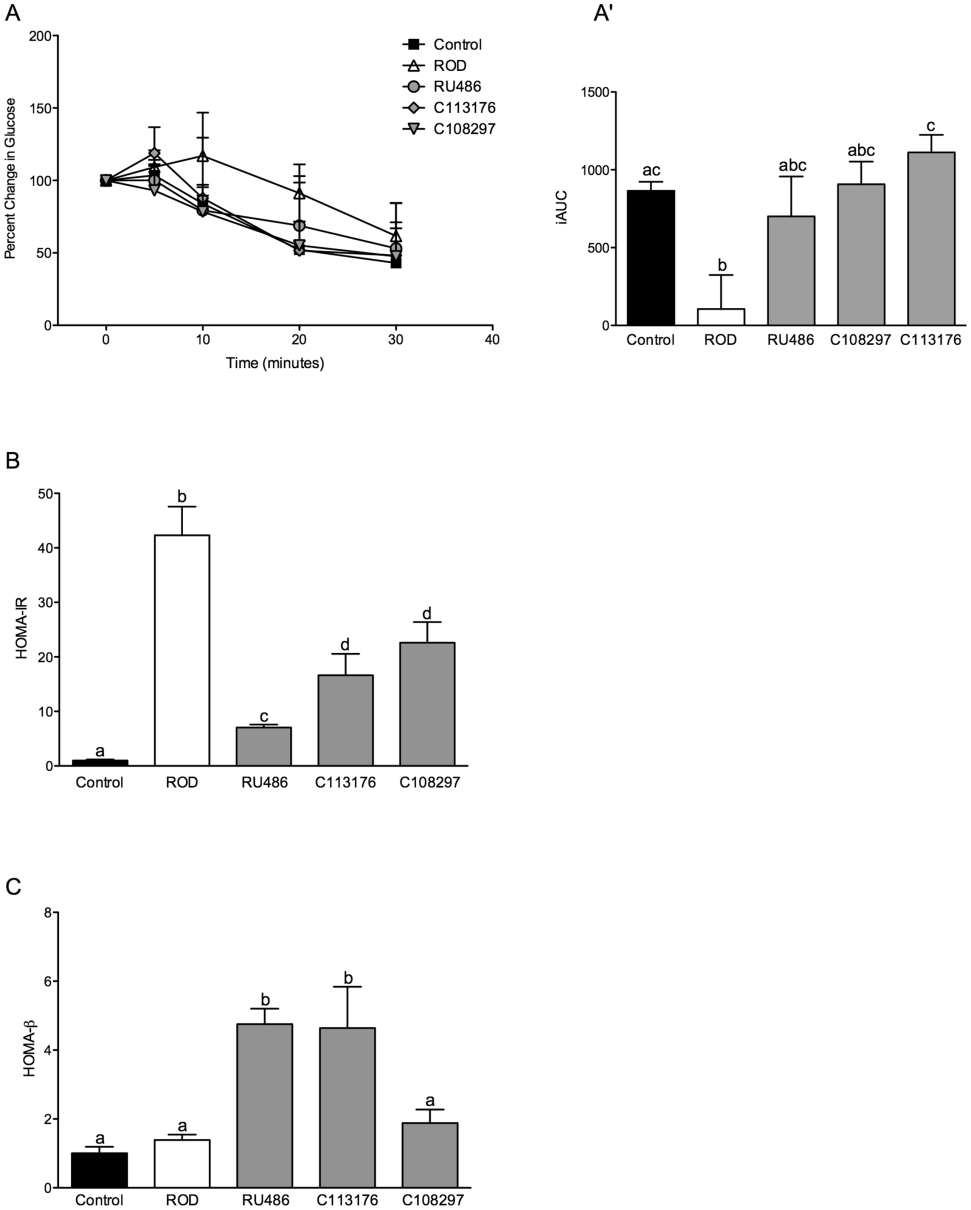
Peripheral insulin sensitivity and β-cell function is enhanced with RU486 and C113176 administration. Percent change of blood glucose levels (mM) relative to individual fasting blood glucose at 5, 10, 20 and 30 minutes post insulin i.p. injection using a handheld glucometer (A). The glucose AUC was measured by the net inverse glucose AUC during the ITT (A′). HOMA-IR index was used to measure whole-body insulin resistance (B). HOMA-β index was used to measure pancreatic β-cell function (C). Bars that do not share similar letters denote statistical significance, p<0.05 one-way ANOVA using Tukey's post-hoc test. n = 7–10. All values are means ± SE.

To determine peripheral insulin sensitivity, primarily through skeletal muscle insulin action, an insulin tolerance test (ITT) was performed on all treatment groups. All of the values were plotted as a percent change from each individual's fasting blood glucose level (Figure 3A), since baseline glucose levels differed among the groups. Inverted glucose AUC was calculated as an index of insulin sensitivity (Figure 3A′). The ROD group had the lowest insulin sensitivity (Figure 3A′), while all three antagonists resulted in normalized insulin sensitivity when compared to controls (Figure 3A′). To determine insulin resistance as measured primarily by liver insulin action, the HOMA-IR index was used. ROD animals were ∼40 and 30-fold more insulin resistant than control animals and RU486 treated groups, respectively (p<0.05, Figure 3B). RU486 treated animals were more insulin resistant than control animals (by∼2-fold). However, they were less insulin resistant than the ROD and the selective GRII antagonist treated groups. C113176 and C108297 treated animals showed attenuated increases in insulin resistance when compared to ROD animals. However, these treatment groups remained more insulin resistant than control animals and RU486 treated animals (p<0.05, Figure 3B). The HOMA-β index was used to determine β-cell function; higher values indicate elevated β-cell response to basal glucose concentrations. No differences were found between ROD animals and control treated animals. Both RU486 and C113176 treatments improved β-cell function by ∼5-fold compared to all other groups (p<0.05, Figure 3C). C108297 treatment did not significantly impact the HOMA-β index. It is important to note that HOMA-β represents basal β-cell function and although ROD animals did not show differences between the controls they do have impaired β-cell response to glucose (Figure 2C), and they clearly do not provide adequate insulin levels to reverse hyperglycemia in either the fasted or fed state.

### Body Composition

Relative visceral fat mass was represented by the measured amount of isolated epididymal fat pad mass divided by body mass. All treatment groups had increased relative visceral fat mass compared to the control group (p<0.05, Table 4). No differences in fat mass were found between the groups treated with the antagonists. Liver mass was also increased in all treatment groups compared to controls (p<0.05) except for animals treated with RU486, in which liver mass was normalized (Table 4). Heart mass was increased with ROD and C108297 treatment and normalized with RU486 treatment. Animals treated with C113176 showed slight improvements compared to control animals (p<0.05, Table 4). It is known that CORT treatment specifically impairs white muscle growth [37]. As expected, ROD animals had lower epitrochlearis mass compared to all other treatment groups (p<0.05). All groups that were administered an antagonist did not show changes in muscle mass relative to the animals in the control group (Table 4). The soleus muscle primarily consists of red, slow twitch muscle fibers and has been previously shown to increase with ROD treatment [29], although the mechanisms for this change remain unknown. ROD animals demonstrated an increase in soleus muscle mass (p<0.05, Table 4) whereas RU486 treated animals only had a slight trend towards an increase in soleus muscle mass compared to control animals (p>0.05, Table 4). There were no differences between tibialis anterior muscle mass among the various treatment groups (Table 4).

**Table 4:** Anthropometric data for epididymal fat pad, liver, heart, epitrochlearis, soleus and tibialis anterior mass (g/kg of body mass).

	Control	ROD	RU486	C113176	C108297
**Epididymal Fat Pad (g/kg)**	15.9±0.7^a^	25.0±1.7^b^	26.3±0.7^b^	26.4±1.6^b^	23.3±1.5^b^
**Liver (g/kg)**	38.3±1.1^a^	74.0±3.5^b^	43.1±2.8^a^	62.8±4.5^b^	73.4±4.8^b^
**Heart (g/kg)**	2.6±0.1^a^	4.1±0.2^b^	2.9±0.1^a^	3.6±0.1^c^	4.0±0.1^bc^
**Epitrochlearis (g/kg)**	0.13±0.01^a^	0.09±0.01^b^	0.13±0.01^a^	0.11±0.01^ab^	0.14±0.02^a^
**Soleus (g/kg)**	0.34±0.01^a^	0.45±0.03^b^	0.38±0.02^ab^	0.41±0.02^b^	0.44±0.02^b^
**Tibialis Anterior (g/kg)**	1.61±0.05^a^	1.51±0.08^a^	1.54±0.04^a^	1.88±0.06^a^	1.55±0.07^a^

Note: Different letters denote statistical significance, p<0.05, n = 6–10. All values are means ± SE.

### Fat Accumulation in Muscle and Liver

**Figure 4:**
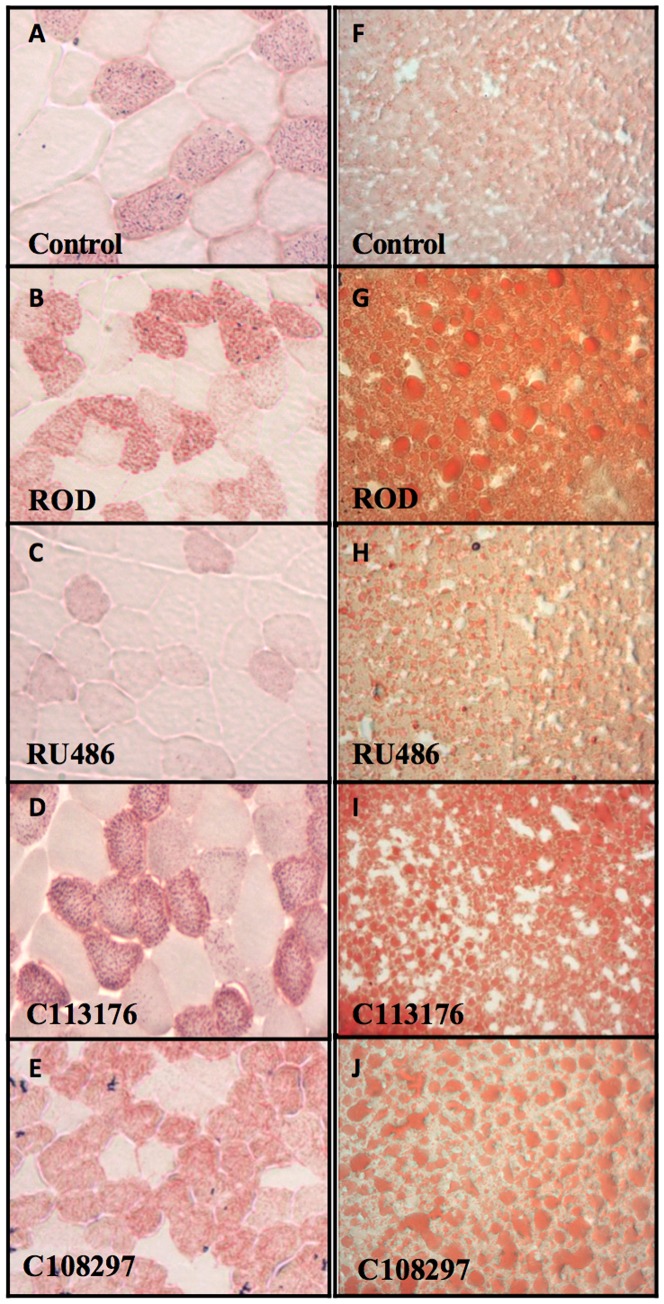
Fat accumulation is normalized with RU486 in skeletal muscle and liver cross sections. To determine fat content in skeletal muscle, tibialis anterior muscle was dissected and stained with a neutral lipid stain (Oil Red O) (A–E). Cross sections of liver were also stained with Oil Red O to measure lipid content (F–J).

Oil Red O staining of liver and tibialis anterior muscle sections was used to quantify fat accumulation in all treatment groups. ROD treatment increased red stain in muscle tissue and appearance of lipid droplets in liver tissue compared to controls (Figure 4). RU486 was the only antagonist that promoted normalized lipid accumulation in both the liver and type IIa muscle fibers relative to controls animals (115.76±10.12 vs. 127.3±4.74, arbitrary units). The animals treated with the selective antagonists (C113176 and C108297) had similar ectopic lipid accumulation compared to ROD treated animals (144.52±3.17, 144.9±2.62, and 142.58±7.44 vs. 141.58±2.98, n = 3–4 per group).

### Protein Content in Visceral Fat

**Figure 5:**
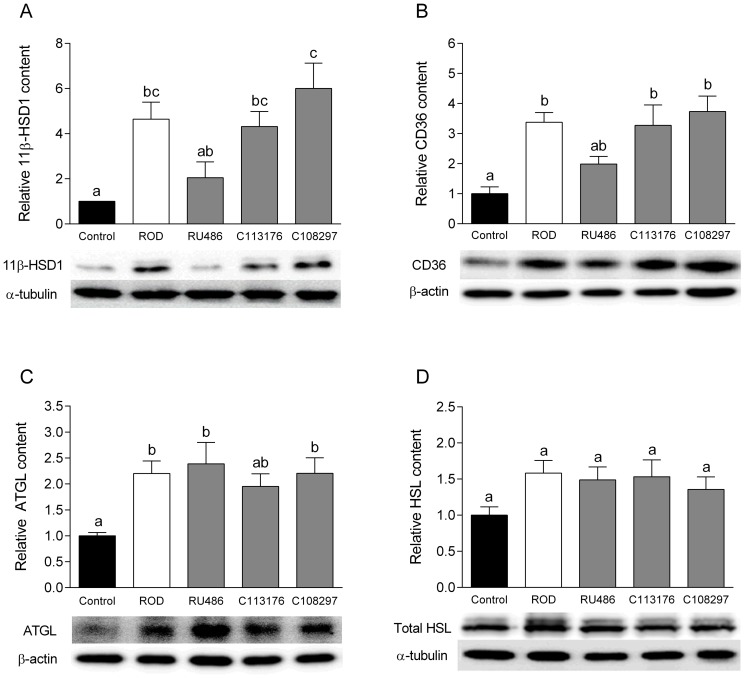
11β-HSD1 content in visceral fat is attenuated with RU486 treatment but not with C113176 or C108297 treatment. No changes were found in lipolytic protein levels with treatment of antagonists. 11β-HSD1 content was measured in epididymal fat pads to represent visceral adipose tissue and expressed relative to α-tubulin content (A). CD36 protein levels were measured from epididymal fat pads (B) as well as adipose triglyceride lipase (ATGL) (C) and hormone-sensitive lipase (HSL) (D) protein levels as markers of lipolytic adipose tissue activity and expressed relative to loading control. Bars that do not share similar letters denote statistical significance, p<0.05 one-way ANOVA using Tukey's post-hoc test. n = 5–6. All values are means ± SE.

At the tissue level, inactive GCs become activated by the action of the pre-receptor enzyme 11β-HSD1, which acts to convert inactive GCs into active GCs. Recent studies have shown that inhibition of 11β-HSD1 activity helps to reverse metabolic pathological conditions such as T2DM [38]. ROD treatment resulted in ∼4-fold increase in 11β-HSD1 content in epididymal fat compared to controls (p<0.05, Figure 5). RU486, but not C113176 nor C108293, treatment normalized levels of 11β-HSD1 content in epididymal fat (p<0.05). CD36 is a protein known to regulate fatty acid uptake into the adipose tissue [39]. ROD treatment increased CD36 protein content by ∼3-fold (p<0.05) but no changes were found in protein content amongst the groups treated with the antagonists (Figure 5B). ATGL and HSL are well known markers of lipolysis that modulate the release of fatty acids from triglyceride storage [35]. ROD, RU486, C113176 and C108297 treated groups, all had increased ATGL protein content compared to controls (all p<0.05), and no differences were found between the antagonist treated groups (Figure 5C). Finally, ROD and all other antagonist treated groups tended to have higher HSL protein levels relative to controls, but these difference from control failed to reach statistical significance (all p>0.05, Figure 5D).

### GSIS (Glucose Stimulated Insulin Secretion)

**Figure 6:**
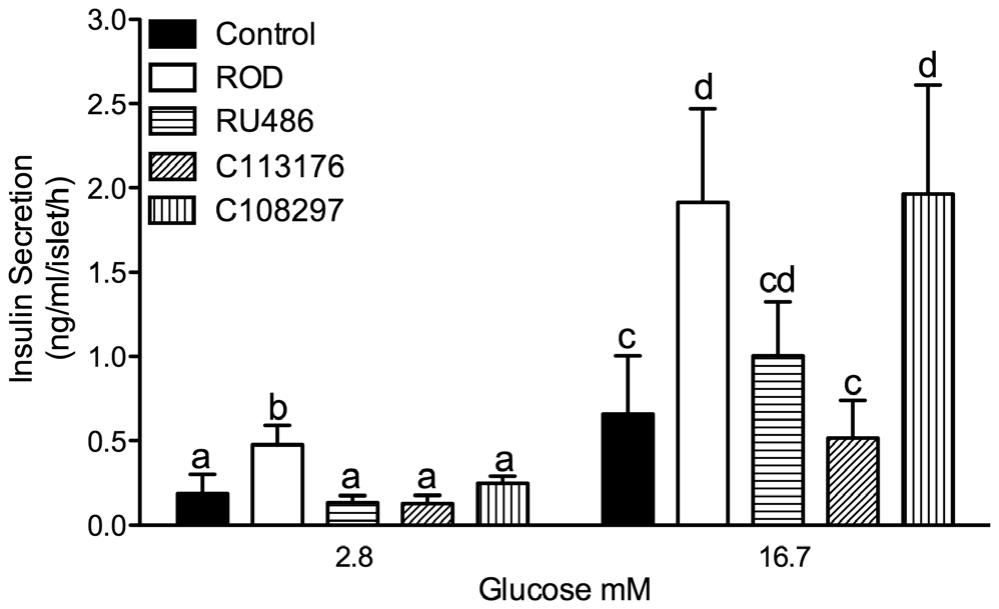
Glucose stimulated insulin secretion (GSIS) was normalized with RU486 and C113176 treatment. GSIS in isolated islets was measured in low (2.8 mM) and high (16.7 mM) glucose media for 1-hour incubations expressed as ng/ml/islet/hour. Bars that do not share similar letters denote statistical significance, p<0.05 one-way ANOVA using student's unpaired t-test. n = 3–7. All values are means ± SE.

Pancreatic islets were isolated from all animals and insulin secretion was stimulated by an exogenous glucose challenge. ROD treatment resulted in an elevation in GSIS in both low (2.8 mM) and high (16.7 mM) glucose media concentrations compared to controls (p<0.05, Figure 6). RU486 and C113176 normalized GSIS in low and high glucose media concentrations compared to controls. C108297 treatment resulted in similar levels as ROD treatment that had higher GSIS in high glucose media compared to control animals (p<0.05) with similar levels of GSIS compared to ROD treated islets.

## Discussion

This study is the first to investigate the effectiveness of selective and non-selective GRII antagonists in a rodent model of ROD induced by GCs and HFD. We show that of the tested compounds, C113176 treatment is the more effective selective GRII antagonist as it attenuates elevated fasting glucose, reinstates β-cell function, and improves insulin resistance. The other selective antagonist tested in this study, C108297, also provides modest attenuation in hyperglycemia and peripheral insulin resistance and no changes to insulin response *in vivo* or β-cell function in this ROD model despite increased *ex vivo* β-cell response. However, by comparison, the non-selective antagonist RU486 demonstrated superior effectiveness in this animal model of hyperglucocorticoidemia/diabetes, completely normalizing all abnormal features of growth and metabolism. Nonetheless, the new selective antagonist C113176 may be of some therapeutic advantage for treatment of the metabolic features of hyperglucocorticoidemia/diabetes since it does not bind to the progesterone receptor.

Elevated GCs induce a severe state of hypercatabolism that can lead to drastic metabolic complications, such as muscle wasting, low bone density and inhibited structural growth [7]. GCs can also act in an anabolic fashion, promoting increased food intake and body fat deposition, especially in the abdominal region, thereby increasing an individual's risk for the development of diabetes [7]. We have previously reported that ROD treatment decreases overall body mass (∼50%) but increases visceral adiposity compared to control animals [29]. In the present study, we show that RU486 treatment reverses body mass loss caused by ROD treatment, increasing body mass gain by ∼20% compared to pre-surgery mass (Fig. 1A and B). Previous results show that RU486 and C108297 treatment lower body mass gain in C57BL/6J mice consuming a HFD and given access to 11% sucrose for 4 weeks [27]. An obese phenotype is also alleviated with RU486 [28], [40]–[42] and C108297 [42] in other rodent and human models of disease. However, to our knowledge, C113176 administration in animals with elevated GCs who are consuming a high fat diet has never before been reported. We demonstrate in this study that C113176 antagonism restores pre-surgery body mass at day 8 of treatment and animals continue to maintain a healthy body mass until the termination of the study. In contrast, C108297 treatment promoted mass loss similar to that observed in untreated ROD animals, a finding that could be considered detrimental in this particular animal model of severe cachexia. In other studies, C108297 has been shown to decrease mass gain in C57BL/6J mice [27] as well as in rats treated with olanzapine, an anti-psychotic medication associated with weight gain in rodents and humans [42]. Body composition analysis was not reported in these studies to determine if the drug influenced lean or fat mass or both. Therefore, C113176 antagonism might be an appropriate treatment for body mass management in humans with Cushing's syndrome because C108297 did not prevent body mass loss.

RU486 has been used to effectively treat metabolic disturbances and hypertension in patients with Cushing's syndrome [24]. It is readily absorbed, has a half-life up to 48 hours, and has a greater binding affinity to GRII than GC agonists such as dexamethasone (3 to 4 times) and cortisol itself (18 times) [43]–[45]. The potent blockade of the GRII with RU486 administration interrupts GC negative feedback to the HPA axis, which could have an effect on corticosterone levels. In our study, there were no differences in CORT AM levels between ROD and antagonist treated animals (Table 2). This finding is not surprising as our model of ROD administers exogenous GCs via CORT pellets at a level that already inhibits HPA axis negative feedback and reduces adrenal GC production [29]. It can therefore be concluded that the results of the antagonists on whole-body metabolism are not due to differences in CORT levels as these were unchanged by the antagonists. It is known that increased levels of stress hormones not only promote hyperphagia [29], but also influence food choice decisions towards higher caloric foods [46]. In this study, ROD treatment resulted in increased relative food intake compared to control animals and the GRII antagonist treated groups. Our study confirms that relative food intake is lower with the administration of GRII antagonists, as has been reported for RU486 treatment in rodents on a HFD [47] and in humans with Cushing's syndrome [24], [40]. In addition, RU486 treatment in obese fa/fa rats with increased levels of circulating GCs, results in lower food intake [48] while no alterations in feeding are found in lean rats given the antagonist [49]. Moreover, studies investigating the effects of selective GRII antagonists on weight gain show no differences in food intake in healthy rodents despite decreases in body mass gain [28]. Taken together, these results suggest that GRII antagonism helps limit hyperphagia in Cushing's syndrome and in diabetic animals.

RU486 administration improves glycemic control and insulin sensitivity in rodents given HFD for 4 weeks [27] and lowers glycated hemoglobin levels in patients with Cushing's syndrome who often exhibit poor glycemic control [24]. In our study, we show that RU486 administration results in normalized glycemic control, and that C113176 lowers fasting glucose better than C108297, but does not improve glucose tolerance compared to ROD treated animals (Figure 2A and A′). Moreover, RU486 results in higher insulin AUC during oral glucose challenge suggesting an elevated insulin response to exogenous glucose, whereas the selective GRII antagonists show no effect on insulin secretion (Figure 2B′). Both RU486 and C113176 treatment resulted in higher AIRs to oral glucose challenge compared to the other treatment groups thereby suggesting that enhanced β-cell function occurs with GRII antagonism (Figure 2C). Animals treated with C108297 tended to have lower AIR compared to controls, suggesting poor β-cell sensitivity to exogenous glucose, similar to the effects seen in ROD animals. In accordance with these findings, we recently have shown that ROD treatment results in impaired islet glucose responsiveness *in vivo* and *ex vivo*, which likely exacerbates the poor glucose control caused by reduced insulin sensitivity [30]. Importantly, this present study suggests that the non-selective GRII antagonist RU486 reverses defective insulin responsiveness *in vivo*, which may help to explain normal glucose control in these treated animals. In addition, β-cell function *in vivo*, as assessed by HOMA-β, is improved with both RU486 and C113176 treatment but not with C108297 treatment (Figure 3C). Moreover, dynamic insulin responses to glucose challenge also appear enhanced by RU486 (Figures 2B′ and C). There are very few studies to date that investigate the effects of RU486 on β-cell function *in vivo* and to the best of our knowledge this study is the first to report on β-cell function *in vivo* with selective GRII antagonism. As such, it is possible to suggest that improvements to β-cell function *in vivo* are through exclusive inhibition of GRII as seen with C113176 administration. However, more investigations are required to fully understand the mechanisms of action with selective GRII antagonism.

Elevated GCs in rodents induce peripheral tissue abnormalities that affect liver and skeletal muscle insulin sensitivity/signalling, thereby promoting hyperglycemia [39], [50], [51]. In our study, we show that RU486, C113176 and C108297 normalize whole body insulin sensitivity (Figure 3A and A′) although C108297 was the only group that was statistically significant from the ROD group. The blockade of the GRII with RU486 helps to reduce peripheral insulin resistance in individuals with mild GC excess [52]. Our study confirms that insulin resistance, as measured by HOMA-IR was normalized with RU486 while attenuated with C113176 and C108297 administration (Figure 3B). Liver and skeletal muscle lipid accumulation is elevated in ROD treated animals, ultimately contributing to peripheral insulin resistance [29], [39]. Interestingly, only RU486 administration lowered both skeletal muscle and liver fat content compared to the ROD animals, with the other selective GRII antagonists having no noticeable effect (Figure 4). Therefore, more studies are required to investigate the mechanisms of action, especially with C113176 administration, on improving insulin resistance.

Patients with Cushing's syndrome have an increased risk of obesity, which heightens their risk (60–80%) of developing diabetes as the disease progresses [13]. Recent research shows that administration of RU486 results in normal body mass gain in rodents and in healthy men [40], [41]. In addition, selective GRII antagonists such as C112716 and C113083 have been shown to help reverse olanzapine-induced mass gain in rats [28]. In our study we found that none of the GRII antagonists normalized the visceral adiposity that is readily observable in ROD treated animals (Table 4). We propose that it is likely that GRII antagonism inhibits GC-induced lipolysis [53], [54] or off-target non-genomic effects that may indirectly increase GC tissue action [55], thereby promoting visceral adiposity. Moreover, increased 11β-HSD1 activity in adipose tissue has been linked to obese and/or insulin resistant individuals [56]. Our ROD model demonstrates increased 11β-HSD1 content in visceral fat compared to controls. RU486 attenuates 11β-HSD1 content (Figure 5), contradicting the idea that an increase in GC activity results in more visceral adiposity as RU486 treated animals had similar visceral adiposity to the ROD treated animals. Therefore, we propose that the increase in visceral adiposity with RU486 is due to the anti-lipolytic effects of increased insulin levels as all ROD treated groups had a higher level of insulin than controls (Table 4). Insulin is an anabolic hormone that down-regulates non-genomic actions of GCs (lipolysis) [54], [57], thereby promoting increased adiposity. In support of this, we found that ROD treatment increases lipolytic enzyme protein content in visceral fat depots and none of the antagonists appeared to normalize these increases (Figure 5 B–D). Together these data suggest that although GRII antagonism does not lower visceral adiposity it may decrease lipolytic GC action in the adipose tissue of RU486 treated rats, in part indicating a lesser risk of developing adiposity-induced abnormalities.

Previously we have shown that ROD treatment results in increased islet 11β-HSD1 content as well as elevated GSIS in isolated islets [30]. RU486 administration to insulin secreting cells *in vitro* reverses β-cell dysfunction through improvements in insulin biosynthesis, release and content [58] as well as β-cell [Ca2+]i response to glucose [59]. Our study confirms these results as we show that RU486 and C113176 normalize GSIS compared to controls whereas C108297 has similar GSIS compared to ROD animals (Figure 6). This is the first study to report the effects of selective GRII antagonists on *ex vivo* islet GSIS. We observe that C113176 is an effective antagonist on GC action in the islet and it normalizes GSIS whereas C108297 does not demonstrate the same result. More studies are required *in vitro* to replicate and further investigate the mechanisms of C113176 action on GSIS.

It is evident that GCs play a critical role in regulating whole body physiology [60] and reducing their action, if concentrations are too elevated, can help eliminate some unwanted effects. Although RU486 binds to the GRII with high affinity (0.3 nM) it also binds with high affinity to the progesterone receptor, resulting in endometrial wall thickening causing early disruption to pregnancy in females (reviewed in, [21]). Therefore, selective GRII antagonists have a major advantage over non-selective antagonists and presently there is a significant effort underway to find replacements for non-selective GRII blockers [61]. More experiments need to be completed before the best antagonist is identified but in our study we consistently see that one compound, C113176, attenuate diabetes symptoms. The advantage of C113176 is that it binds to the GRII with excellent affinity (0.28 nM) and does not bind significantly to ER, AR, PR or MR (Table 1). It has been found to be more potent than C108297 in functional TAT (tyrosine amino transferase) *in vitro* assays that measure inhibition of dexamethasone-induced activity in HepG2 cells and ratH4 cells (Corcept Therapeutics Inc.). For example, in HepG2 cells, the Ki values for C108297 and C113176 are 25 and 11 nM, and in rat H4 cells, the Ki values are 14 and 4 nM, respectively (Table 1). Compounds such as C108297 are described as having some beneficial effects in inhibiting GC action. However, C108297 does not completely reverse adverse GC effects in our ROD model. Perhaps, as previously described [26], this antagonist may actually work as a partial agonist, which could account for some of the results presented.

This study describes the various metabolic and morphologic outcomes of administering selective and non-selective GRII antagonists to our rodent model of ROD. We show that in comparison to RU486, C113176 provides therapeutic advantages over C108297 in ROD animals. C113176 helps to attenuate fasting hyperglycemia, insulin resistance and improves AIR, and pancreatic β-cell response. It may be a reasonable alternative medication to help patients suffering from Cushing's syndrome and other diseases associated with elevated GCs. Although C113176 did not completely normalize all complications of diabetes, it did provide clear benefits without any observable adverse effects. Thus, C113176 may be a good alternative to the non-selective GRII antagonist RU486.
